# DRJAMM Is Involved in the Oxidative Resistance in *Deinococcus radiodurans*

**DOI:** 10.3389/fmicb.2021.756867

**Published:** 2022-01-28

**Authors:** Jianling Cai, Chaoming Pan, Ye Zhao, Hong Xu, Bing Tian, Liangyan Wang, Yuejin Hua

**Affiliations:** Ministry of Education Key Laboratory of Biosystems Homeostasis and Protection, Institute of Biophysics, College of Life Sciences, Zhejiang University, Hangzhou, China

**Keywords:** *Deinococcus*, JAMM/MPN^+^, deubiquitinase, antioxidation, DMSO

## Abstract

Proteins containing JAB1/MPN/MOV34 metalloenzyme (JAMM/MPN^+^) domains that have Zn^2+^-dependent deubiquitinase (DUB) activity are ubiquitous across among all domains of life. Recently, a homolog in *Deinococcus radiodurans*, DRJAMM, was reported to possess the ability to cleave DRMoaD-MoaE. However, the detailed biochemical characteristics of DRJAMM *in vitro* and its biological mechanism *in vivo* remain unclear. Here, we show that DRJAMM has an efficient *in vitro* catalytic activity in the presence of Mn^2+^, Ca^2+^, Mg^2+^, and Ni^2+^ in addition to the well-reported Zn^2+^, and strong adaptability at a wide range of temperatures. Disruption of *drJAMM* led to elevated sensitivity in response to H_2_O_2_ *in vivo* compared to the wild-type R1. In particular, the expression level of MoaE, a product of DRJAMM cleavage, was also increased under H_2_O_2_ stress, indicating that DRJAMM is needed in the antioxidant process. Moreover, DRJAMM was also demonstrated to be necessary for dimethyl sulfoxide respiratory system in *D. radiodurans*. These data suggest that DRJAMM plays key roles in the process of oxidative resistance in *D. radiodurans* with multiple-choice of metal ions and temperatures.

## Introduction

Proteins containing JAMM/MPN^+^ domain (JAMMs) have been found in prokaryotes, eukaryotes, and archaea. They play important roles in all kinds of cellular processes such as DNA repair (Zeqiraj et al., 2015), pre-mRNA-processing (Galej et al., 2014), and sulfur mobilization to form molybdenum cofactors (Cao et al., 2015). Generally, JAMMs cleave the ubiquitin-like small archaeal modifier proteins (SAMP1/2) or MoaD-MoaE in the presence of Zn^2+^, for instance, HvJAMM1 (Hepowit et al., 2012), PfHAMM1 (Cao et al., 2017), and CSN5 (Altmann et al., 2017). It is worth noting that the MPN domain super-family has two main subclasses: MPN^+^ and MPN^–^. The MPN^+^ domain-containing proteins are zinc-dependent isopeptidases with the conserved sequence (E-x[2]-H-S/T-H-x[7]-S-x[2]-D) (Cope et al., 2002; McCullough et al., 2004; Moretti et al., 2010). The functional activity of zinc-dependent isopetidases involves zinc bound to the proteins via two histidines and one aspartic acid residues, such as AMSH (Davies et al., 2011) and CSN5 (Echalier et al., 2013), which are JAMM/MPN^+^ proteins and have the similar organization and composition. The proteins of the MPN^–^ family lack catalytic activity due to the absence of pivotal residues in the typical JAMM motif and are usually found in pairs in multi-protein complexes with JAMM^+^ proteins. For example, the eIF3 and COP9 complex has eIF3f and CSN5 representatives of the JAMM/MPN^+^ family and MPN^–^ family members, namely eIF3h and CSN6 (Zhou et al., 2008; Sharon et al., 2009).

After decades of research, it has been found that the action of deubiquitinating enzymes or DUBs controls most ubiquitination events dynamically (Amerik and Hochstrasser, 2004). In addition, the deubiquitination process is achieved by hydrolyzing the last residue of the isopeptide bond after Gly76 or the peptide bond of the polyubiquitin chains connected to Met1 (Wilkinson, 1997; Love et al., 2007). According to the structural analysis of the active domain, DUBs can be divided into five subfamilies: the Ub C-terminal hydrolases (UCHLs), the Ub-specific proteases (UBPs), ovarian tumor proteases (OTUs), the Josephin domain proteases (JDs), and JAB1/MPN/MOV34 (JAMMs) (Nijman et al., 2005). For example, in *Haloferax volcanii*, HvJAMM1 can cleave proteins attached to SAMP1 by linear and isopeptide bonds, and the C-terminal diglycine motif of SAMP1 is not required for HvJAMM1 mediated-cleavage of linear protein fusions (Hepowit et al., 2012). In *Pyrococcus furiosus*, the PFJAMM1 can identify SAMP2 with accuracy, regardless of the target protein connected to the C-terminal Gly of the SAMP2 (Cao et al., 2017). In eukaryotes, AMSH is demonstrated to have DUB activity (Kyuuma et al., 2006).

*Deinococcus radiodurans* is well-known for its powerful capacity to endure extreme stresses such as ionizing radiation (IR), desiccation, and oxidation (Makarova et al., 2001; Daly, 2012). Studies demonstrated that oxidative stress is incurred by reactive oxygen species (ROS) (Goswami et al., 2006). The antioxidant defense mechanism of *D. radiodurans* is active against all three main ROS, including hydroxyl radicals (OH⋅), superoxide radicals (O_2_⋅^–^), and hydrogen peroxide (H_2_O_2_). To remove the dangerous ROS and adapt to the oxygen-rich environment of Earth, *D. radiodurans* has evolved a variety of mechanisms to cope with stressful situations. For example, MnSOD (DR1279), a superoxide dismutase (SOD) of *D. radiodurans*, scavenges the superoxide more efficiently than its homologs in humans and *Escherichia coli* due to a more rapid protonation and release of H_2_O_2_ (Abreu et al., 2008). As reported, *D. radiodurans* contains a high concentration of manganese and keeps high intracellular total manganese to total iron ratio of 0.24 compared to that of radiation-sensitive bacteria (< 0.01 in *E. coli*) (Daly et al., 2004). And *D. radiodurans* contains three eukaryotic-type catalases, which are constitutively expressed in normal conditions (Lipton et al., 2002; Jeong et al., 2016). These findings give us insights into understanding the oxidative damage response mechanisms in *D. radiodurans*, while numerous genes related to oxidative resistance in *D. radiodurans* have not been clearly studied in detail (Yang et al., 2014).

Recently, *dr_0402* has been found to encode the JAMM/MPN protein, and the product of its expression, DRJAMM, could cleave the MoaD-MoaE fusion protein (DR2607) and generate a C-terminal Gly residue (Yang et al., 2018). MoaD-MoaE is known as the MPT synthase that catalyzes the formation of MPT from cyclic pyranopterin monophosphate (cPMP) converted from 5′-GTP, while the two sulfur molecules on cPMP are carried as thiosulfates on the C-terminal glycine of MoaD (Leimkuhler et al., 2001; Zupok et al., 2019). During the formation of MPT, the substrate pocket of MoaE can bound the cPMP, MPT, and the C-terminal of MoaD. It has been shown that the utilitarian action of MoaD-MoaE as an MPT synthase must be cut-activated by JAMMs (Cao et al., 2015; Narrandes et al., 2015). In addition, MoaD and MoaE are essential for molybdenum cofactor (Moco)-dependent dimethyl sulfoxide (DMSO) reductase activity in archaea (Miranda et al., 2011). However, only one pair of DRMoaD-MoaE fusion protein is encoded in *D. radiodurans*, and the detailed catalytic activity of DRJAMM *in vitro* and its biological significance *in vivo* are still unknown.

In the present study, we found that DRJAMM could efficiently cleave DRMoaD-MoaE not only in the presence of Zn^2+^ but also in the presence of other metal ions *in vitro* under either low or high temperatures. Meanwhile, mutation of *drJAMM* led to a decreased survival rate and elevated transcriptional levels of DMSO reductase in response to H_2_O_2_ *in vivo* compared to the wild-type R1, indicating that DRJAMM plays an important role in the antioxidant process of the organism.

## Results

### DRJAMM Cleaving Activity Is Dependent on a Variety of Metal Irons

In previous study, MoaD-MoaE has been shown to play an irreplaceable role in the transformation of cyclic pyranopterin monophosphate (cPMP) into molybdopterin (MPT) in *E. coli* (Neumann et al., 2009). To confirm the importance of DRJAMM to MPT synthase, cPMP was oxidized into its stable fluorescence derivatives, compound Z (Wuebbens and Rajagopalan, 1995; Dahl et al., 2013). When the amount of cPMP was set to 100% in *drJAMM* mutant strain, it was not detected in wild-type R1 strain and *drJAMM* complementary strain (Supplementary Figure 1), indicating that DRJAMM is essential for the activation of DRMoaD-MoaE.

**FIGURE 1:**
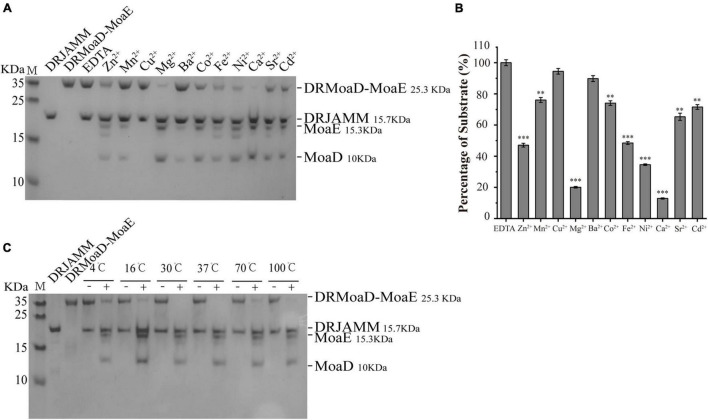
The metal ion preference and temperature adaptability of DRJAMM activity. **(A)** Analysis of ion effects on DRJAMM function. The reaction of 10 μM DRMoaD-MoaE and 40 μM DRJAMM was incubated at 37°C for 30 min with 0.4 mM metal ions. Products were separated with Tricine-SDS-PAGE. **(B)** Values are the means of three independent assays (mean ± SD), **p < 0.01, ***p < 0.001. **(C)** Analysis of temperature effects on DRJAMM function. Reactions were conducted similar to panel A but with the temperature gradient increased from 4 to 100°C. “−” represents EDTA control, “ + “ represents Ca^2+^ treatment.

It has been previously revealed that DRJAMM requires Zn^2+^ to cleave the MoaD-MoaE fusion protein (Yang et al., 2018). However, we found that other metal ions could also catalyze this cleavage activity *in vitro*, such as Mn^2+^, Mg^2+^, Ca^2+^, and Ni^2+^ (Figure 1A). The remaining DRMoaD-MoaE fragments were quantified using ImageJ software (National Institutes of Health, United States) to demonstrate the catalytic efficiency of DRJAMM in the presence of different metal ions, as shown in Figure 1B. Unexpectedly, DRJAMM displayed the highest catalytic efficiency in the presence of Ca^2+^, about threefold higher than that of Zn^2+^ (Figure 1B). In addition, high temperatures did not inhibit the activity of JAMMs. Since the catalytic function of PfJAMM1 was the best at 100°C in *Pyrococcus furiosus* (Cao et al., 2017), we set a series of temperature gradients and found that the DRJAMM exhibits catalytic activities at different temperatures. Surprisingly, DRMoaD-MoaE is stably degraded by DRJAMM even under high temperatures (Figure 1C). These results suggested that the enzyme activity of DRJAMM has strong adaptability to a wide range of temperatures, even above 100°C.

### *drJAMM* Is Involved in the Antioxidative Process

**FIGURE 2:**
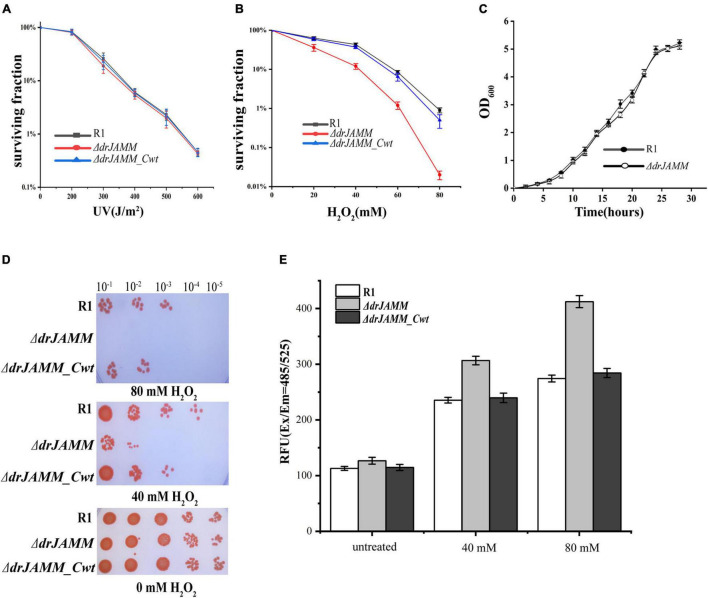
Phenotypes of the *D. radiodurans* wild-type strain (R1), the mutant Δ*drJAMM* (Δ*dr_0402*), and Δ*drJAMM* compensatory stain (Δ*dr_0402_Cwt)*. **(A,B)** Survival curves of the strains under H_2_O_2_ (0–80 mM) and UV (0–600 J/m^2^) treatment, respectively. **(C)** Growth curves of *D. radiodurans* wild-type strain and Δ*drJAMM* mutant strain. The data represent the means of the three replicates. **(D)** Following incubation with 0, 40, or 80 mM H_2_O_2_ for 30 min, the strains were spotted onto TGY plates. The numbers above the figure represent the dilution ratio of cultures. **(E)** The level of ROS accumulation in cells after 0, 40, and 80 mM H_2_O_2_ treatment, respectively. “Untreated” represents a concentration of 0 mM H_2_O_2_. RFU means relative fluorescence units.

To investigate the function of DRJAMM in *D. radiodurans*, a *drJAMM* (Δ*dr_0402*) knockout mutant was constructed and the cell survival rate and cell growth curves were measured (Figures 2A–C). It was shown that the mutant Δ*drJAMM* was not sensitive to UV radiation but declined significantly under H_2_O_2_ (0–80 mM) than the R1 (wild-type). The sensitivity is nearly disappeared after complementation with *drJAMM* (Δ*dr_0402_Cwt*) in the mutant. However, the Δ*drJAMM* growth curve displayed no change during a stationary phase of approximately 30 h (Figure 2C), indicating that the *drJAMM* mutation does not influence the growth rate of *D. radiodurans* but affects its response to oxidative stress.

Further analysis of oxidative stress survival with a spot-test method also demonstrated that the mutant is highly sensitive to H_2_O_2_, and could not endure 80 mM H_2_O_2_, but could be recovered after gene complementation (Figure 2D). Hence, the ROS level was measured to verify the role of *drJAMM* in the antioxidant process of *D. radiodurans*. As shown in Figure 2E, the ROS accumulation level in mutant was about 1.3-fold than that of R1 following 40 mM H_2_O_2_ treatment, while 1.5-fold higher after 80 mM H_2_O_2_ treatment. Furthermore, the ROS level in the mutant rises with the increase in H_2_O_2_ concentration, which was found to be restored to wild-type levels in the complementary strain. This suggests that the absence of *drJAMM* will cause the accumulation of ROS. Therefore, *drJAMM* is critical for the antioxidation process in *D. radiodurans*.

### Levels of DRJAMM and DRMoaD-MoaE Increase Under Oxidative Stress

**FIGURE 3:**
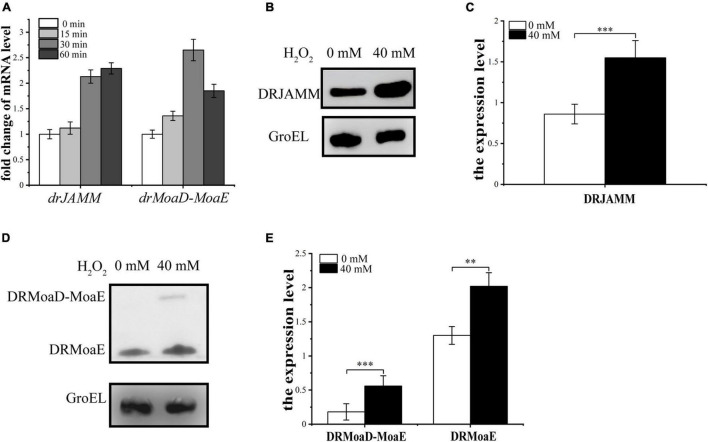
Analysis of transcriptional and expressional levels of *drJAMM* and *drMoaD-MoaE* under oxidative stress. **(A)** The mRNA levels of *drJAMM* and *drMoaD-MoaE* after exposure to 40 mM H_2_O_2_ for 15, 30, and 60 min. **(B,C)** The expression level of DRJAMM in the presence or absence of H_2_O_2_. GroEL was used as a control, and an anti-GroEL antibody was used for detection. The relative band strength was scanned and quantified from three independent experiment using ImageJ software. The expression level of each protein was normalized based on the expression level of GroEL, **p < 0.01, ***p < 0.001. **(D,E)** The expression level of DRMoaD-MoaE and DRMoaE in the presence or absence of H_2_O_2_. GroEL was used as a control, and an anti-GroEL antibody was used for detection. The expression level of each protein was normalized based on the expression level of GroEL. Values were means of three independent assays (mean ± SD), **p < 0.01, ***p < 0.001.

To test the cleavage efficiency of DRJAMM (DR0402) to DRMoaD-MoaE (DR2607) during antioxidant processes, a His-tag was fused to the C-terminal of DRJAMM and DRMoaD-MoaE *in situ*. The transcriptional and expressional levels of *drJAMM* and *drMoaD-MoaE* were analyzed using qRT-PCR and western blot assays following H_2_O_2_ treatment in the wild-type R1 strain. The mRNA levels of *drJAMM* and *drMoaD-MoaE* are increased under H_2_O_2_ treatment (Figure 3A), suggesting that they both may be involved in the oxidative resistance of *D. radiodurans*. Similarly, western blot assays showed that the expression of DRJAMM and DRMoaD-MoaE are both remarkably elevated about 1.5-fold and 3-fold, respectively, following H_2_O_2_ treatment, while the expression level of DRMoaE is also increased significantly about 1.5-fold (Figures 3B–E), indicating that the cleavage activity of DRJAMM might be necessary for oxidative resistance.

### DRJAMM Is Required for Dimethyl Sulfoxide Respiration System

DMSO reductase activity is dependent on molybdenum cofactor (Moco) synthesis that requires JAMM/MPN metalloprotease (Miranda et al., 2011). Sequence alignment suggested that *dr_0397* encode a molybdopterin oxidoreductase that has been shown to play a role in dimethyl sulfoxide respiration in *Rhodobacter capsulatus* (Solomon et al., 2000), and is homologous to *E. coli* DMSO reductase (Supplementary Figure 2).

**FIGURE 4:**
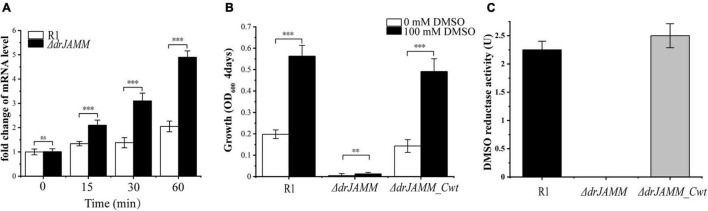
Determination of levels of DMSO reductase under oxidative stress, growth of strains under anaerobic conditions, and the activity of DMSO reductase. **(A)** The mRNA levels of the DMSO reductase at different times following exposure to 40 mM H_2_O_2_ in R1 and Δ*drJAMM.* Values were means of three independent assays (mean ± SD), *ns*, not significant, ***p < 0.001. **(B)** Anaerobic growth of R1, Δ*drJAMM* and Δ*drJAMM_Cwt* in medium containing 0 or 100 mM DMSO for 4 days. Values were means of three independent assays (mean ± SD), **p < 0.01, ***p < 0.001. **(C)** The DMSO reductase activity was monitored with nitrogen at A600 nm. The U was defined as 1 μmol substrate consumed per minute at room temperature. Values were means of three independent assays (mean ± SD).

To confirm whether the absence of *drJAMM* will affect the DMSO respiration system in *D. radiodurans*, the transcriptional levels of DMSO reductase were measured using qRT-PCR. Compared with wild-type R1, the mRNA levels of the DMSO reductase are more strongly elevated in Δ*drJAMM* following exposure to H_2_O_2_, though the levels are also induced in R1 (Figure 4A), suggesting deletion of *drJAMM* causes a large demand for DMSO reductase in the antioxidant process. In the absence of DMSO, the growth of all strains was inhibited, while the addition of DMSO restarted growth. However, the growth of Δ*drJAMM* is still in stagnation after adding DMSO (Figure 4B), indicating that *drJAMM* is necessary for the DMSO respiration system. In *Haloferax volcanii*, the JAMM/MPN^+^ metalloprotease HvJAMM1 can activate MPT synthase, and anaerobic growth using DMSO as a terminal electron acceptor can be used as a method to monitor the activation of MPT synthase by HvJAMM1 (Hepowit et al., 2012).

To further investigate the function of DRJAMM in the DMSO respiration system, the wild-type R1, *drJAMM* mutant strain, and *drJAMM* complementary strain were grown to OD_600_ = 1.0 under aerobic conditions, and then incubated with DMSO under anaerobic conditions. The DMSO reductase activity was not detected in the cell lysate of *drJAMM* mutant strain, but could be readily detected in wild-type R1 and *drJAMM* complementary strain (Figure 4C), suggesting that DRJAMM is important for the maturation of DMSO reductase protein.

## Discussion

A broad spectrum of species encoding JAMM/MPN domain proteins are dependent on Zn^2+^. For instance, the activity of HvJAMM1 can be activated by the addition of excess ZnCl_2_ (Hepowit et al., 2012), and the loss of structural zinc leads to a significant reduction in the thermal stability of AMSH (Bueno et al., 2015). However, in the present study, the JAMM/MPN + protein DRJAMM could be activated by different metal ions such as Mn^2+^, Mg^2+^, Ca^2+^, and Ni^2+^ besides Zn^2+^. Interestingly, more and more multi-metal-dependent nucleic acid enzymes (NAE) have been found to choose sulfophilic metal based on the characteristics of the reaction, or to perform the response through polymetallic collaboration (Zhou and Liu, 2018). Hence, as to how different metal particles control the action of DRJAMM amid the antioxidant handle, and whether there are numerous administrative components like multi-metal-dependent NAE needs further structural explanation.

Usually, proteins will be denatured and lose their function at high temperatures (Bischof and He, 2005). However, DRJAMM displayed stable protease activity even at 100°C. From the perspective of genome evolution, it is proposed that *D. radiodurans* has obtained many genes from *Thermus thermophilus* (Makarova et al., 2001), which may explain the resistance of high temperatures by DRJAMM.

A previous study revealed that HvJAMM1 regulates sumoylation and HvJAMM1-type proteins are thought to release SAMP (Hepowit et al., 2012). Meanwhile, DRJAMM contains a conserved motif similar to HvJAMM1 (Yang et al., 2018), and has the same reaction product MoaE (Hepowit et al., 2012). MoaE usually forms the MPT synthase with MoaD that shares a common globular β-grasp fold with Ub (Narrandes et al., 2015; Yang et al., 2018). For example, TtuB is a bacterial ubiquitin-like protein that has a similar globular β-grasp fold to the Ub of archaea (Shigi, 2012). In addition, DRMoaE is also associated with the function of ubiquitin-like (Ubl) proteins (Humbard et al., 2010). We speculated that Ubl protein modification system may exist in *D. radiodurans* and DRJAMM might play an important role.

Previous studies showed that BRCC36 is a JAMM (JAB1/MPN/Mov34 metalloenzyme) domain DUB enzyme and is involved in the DNA damage response (Patterson-Fortin et al., 2010). Although the survival rate of Δ*drJAMM* is identical to R1 under UV stress (Supplementary Figure 3), the growth of this mutant is dramatically inhibited relative to the wild-type R1 under H_2_O_2_ stress. Simultaneously, the mRNA level of DRJAMM is significantly increased under oxidative stress, implying the importance of this protein in improving oxidation resistance.

According to previous studies, MoaD-MoaE in molybdenum cofactor (Moco) biosynthesis was able to catalyze some redox reactions *in vivo* (Zupok et al., 2019). In the present study, we found the expression levels of DRMoaE and DRJAMM are increased simultaneously under oxidative stress, indicating that DRJAMM is necessary for DRMoaE activation. A recent study revealed that HvJAMM1 plays an important role in releasing MoaE in Moco biosynthesis through deubiquitination (Cao et al., 2015). Therefore, DRJAMM might participate in the antioxidant process by cleaving the DRMoaD-MoaE fusion protein to release MoaE in *D. radiodurans*.

As an essential enzyme in the Moco biosynthesis pathway of bacteria, MoaE is located upstream of MobB, while MobB is responsible for forming molybdenum guanine dinucleotide commonly found in the DMSO reductase family (McLuskey et al., 2003; Miranda et al., 2011). The SAMP1-MoaE is ineffective in DMSO respiration, and this process requires metalloprotease HvJAMM1 (Cao et al., 2015). Under anaerobic conditions, wild-type R1 and complementary strains can remain in abnormal growth, while growth of the *drJAMM* knockout strain is almost completely stopped. After supplement with DMSO, the mutant still showed weak growth, while both wild-type R1 and the complementary strain recovered. Furthermore, the DMSO reductase activity is nearly completely lost in the *drJAMM* mutant strain. These results verified that *drJAMM* is necessary for the DMSO respiratory system in *D. radiodurans*.

In addition, a variety of microorganisms grow through the respiration of DMSO as an electron acceptor, and several DMSO respiratory systems with different compositions have been identified (Miralles-Robledillo et al., 2019). In a previous study, the thioredoxin (Trx) system, which is composed of NADPH, thioredoxin reductase (TrxR), and thioredoxin, provides the electrons to thiol-dependent peroxidases (peroxiredoxins) to remove ROS, and contributes to the resistance toward oxidative stress in *D. radiodurans* (Holmgren, 2000). Here, the levels of DMSO reductase in *D. radiodurans* are gradually increased under oxidative stress, especially in *drJAMM* knockout strains, implying that DMSO respiratory systems might be involved in the oxidation resistance similar to the Trx system, and *drJAMM* could play an important role in this process.

**FIGURE 5:**
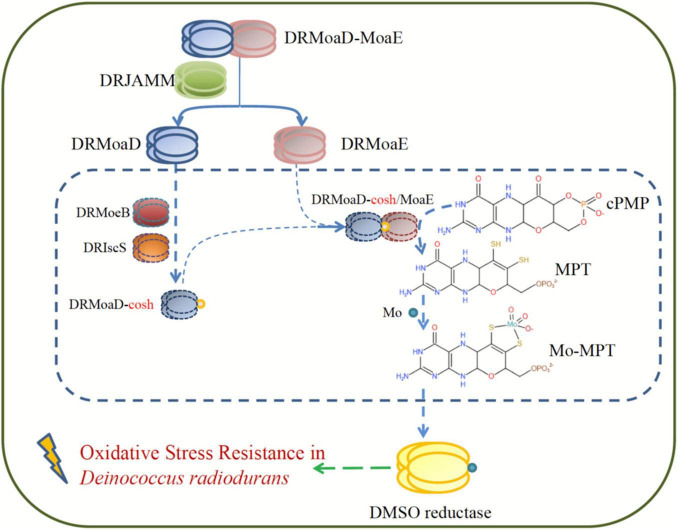
A model of resistance to oxidative stress involving DRJAMM in *D. radiodurans*. Upon oxidative stress, DRMoaD-MoaE is cleaved by DRJAMM to produce DRMoaE and activates the DMSO reductase that participates in the antioxidant process of *D. radiodurans* through the Moco biosynthesis pathway.

Taken together, DRJAMM is essential for resistance to oxidative stress and the DMSO respiration system in *D. radiodurans* (Figure 5). When oxidative damage is encountered, DRMoaD-MoaE is cleaved by DRJAMM to produce DRMoaE, which ultimately affects the DMSO reductase involved in the antioxidant process as demonstrated in *E. coli* (Leimkuhler, 2020). Overall, our findings provide new insights into the role of the JAMM/MPN domain proteins DRJAMM, which can accommodate a multiple-choice of metal ions and temperatures in *D. radiodurans* under oxidative stress.

## Materials and Methods

### Strains and Growth Conditions

All strains, plasmids and primers used in this study are listed in Supplementary Tables 1, 2. *E. coli* strains were grown in Luria-Bertani (LB) liquid medium (1% tryptone, 0.5% yeast extract, and 1% sodium chloride) or on agar (1.5% Bacto-agar) plates supplemented at 37°C with appropriate antibiotics. All *D. radiodurans* strains were grown at 30°C in tryptone glucose yeast extract (TGY) liquid media or on agar plates (0.5% tryptone, 0.1% glucose, and 0.3% yeast extract) supplemented with appropriate antibiotics.

### Expression and Purification of Proteins

The *dr_0402* and *dr_2607* genes were amplified and cloned into a modified pET28a expression vector at *Nde*I and *Bam*HI site (Supplementary Table 2), respectively (Li et al., 2019). Then, the constructed plasmids were transformed into *E. coli* BL21 (DE3), and induced in LB medium containing 50 μg/mL kanamycin and 0.4 mM isopropyl-β-D-thiogalactopyranoside (IPTG) at 30°C for 5 h. Cells were collected and resuspended in lysis buffer (20 mM Tris–HCl, pH 8.0; 500 mM NaCl; 5% (w/v) glycerol; 3 mM β-mercaptoethanol and 9 mM imidazole), followed by sonication. After centrifugation at 20,000 g for 30 min at 4°C, the supernatant was purified using a Ni-NTA column (1 mL, GE Healthcare Biosciences, United States) equilibrated with buffer A (20 mM Tris–HCl, pH 8.0; 500 mM NaCl; 5% (w/v) glycerol and 3 mM β-mercaptoethanol), and washed by buffer B (20 mM Tris–HCl, pH 8.0; 500 mM NaCl; 5% (w/v) glycerol; 3 mM β-mercaptoethanol; 500 mM imidazole). Finally, the proteins were concentrated and purified using a Superdex75 column (GE Healthcare Biosciences, United States), DRJAMM was eluted with buffer C (20 mM Tris–HCl, pH 8.0; 200 mM KCl; and 1 mM EDTA), DRMoaD-MoaE was eluted with buffer D (20 mM Tris-HCl, pH 8.0; and 200 mM KCl).

### DRJAMM Activity Assays

The DRJAMM (DR0402) activity assays were performed as described previously with minor modifications (Yang et al., 2018). The 40 μM of DRJAMM and 10 μM of DRMoaD-MoaE were added into the reaction buffer (100 mM KCl; 20 mM Tris-HCl, pH 8.0; and 1 mM dithiothreitol), and then 0.4 mM of different metal ions were added into samples. The reactions with 0.4 mM Ca^2+^ were incubated at 4, 16, 30, 37, 70, and 100°C for 30 min, respectively, and quenched by the addition of SDS loading buffer followed by immediate boiling. The products were identified by Tricine-SDS-PAGE (12%).

### Construction of Mutant Strains

The mutant strains were constructed by a tripartite ligation method, as described previously (He et al., 2020). Briefly, the DNA fragment upstream of *dr_0402* was amplified by PCR using the primers Δ*dr_0402*-p1 and Δ*dr_0402*-p2, which was digested with *Bam*HI (Supplementary Table 2). The DNA fragment downstream of *dr_0402* was amplified by PCR using the primers Δ*dr_0402*-p3 and Δ*dr_0402*-p4, which were digested with *Hin*dIII (downstream) (Supplementary Table 2). The digested fragment was connected to a streptomycin resistance gene. After the triplet ligation product was transformed into the *D. radiodurans* wild-type R1 strain, the mutant colonies were then selected on TGY plates containing 10 μg/ml streptomycin, and confirmed by genomic PCR using primers Δ*dr_0402*-p5 and Δ*dr_0402*-p6, and DNA sequencing. For complementary strain construction, the wild type *dr_0402* was amplified by PCR using Δ*dr_0402_Cwt*-F and Δ*dr_0402_Cwt*-R, and cloned into the plasmid pRADK containing the *D. radiodurans* groEL promoter; and then transformed into the Δ*dr_0402* mutant strain to obtain the complementary strain Δ*dr_0402_Cwt*.

### Western Blot Analysis

Western blotting was used to confirm protein expression levels were performed as described previously (Dai et al., 2018). The 6 × His-tag was fused to the C-terminus of DRJAMM and DRMoaD-MoaE using tripartite ligation and a double-crossover recombination method. Mouse anti-6 × His tag (Proteintech, United States) was used to detect DRJAMM, DRMoaD-MoaE, and MoaE in the strains. The pre-stained marker was used as reference (Thermo Fisher, United States). The expression level of GroEL was detected using a rabbit anti-GroEL polyclonal antibody (Sigma, United States) in *D*. *radiodurans*, which was used as the internal control.

### Real-Time Quantitative PCR

Real-time quantitative PCR (qRT-PCR) was used to measure *dr_0402* and *dr_2607* gene expression under oxidative stress, as described previously (Dai et al., 2020). First, *D*. *radiodurans* cells were grown to OD_600_ = 1.0 and treated with 40 mM H_2_O_2_ for 30 min. Then, the cells were collected by centrifugation at 5,000 g for 3 min at 4°C. Total RNA was extracted from 5 mL cell cultures using TRIZOL reagent (Invitrogen, Carlsbad, CA, United States). The qRT-PCR experiments were performed using SYBR Premix Ex Taq (TaKaRa Biotechnology, Japan). The primers used in this experiment are listed in Supplementary Table 2. The data were collected and the difference in relative transcription abundance level was calculated. Glyceraldehyde 3-phosphate dehydrogenase (GADPH) encoded by the gene *dr_1343* was used as the internal control.

### Survival Curves, Growth Curves, and Phenotypic Analyses

To measure the survival curves and observe phenotypes under H_2_O_2_ treatment, the wild-type *D. radiodurans* R1 and Δ*dr_0402* were grown to OD_600_ = 1.0, and then treated with different concentrations of H_2_O_2_ for 30 min. After the reaction, the residual H_2_O_2_ was cleared away by adding excess catalase, and then the sample was plated on TGY plates. All the experiments were repeated three times. To measure the growth curve, the wild-type *D. radiodurans* R1 and Δ*dr_0402* were cultured to OD_600_ = 1.0 at 30°C and then 500 μl was transferred into 100 ml of fresh TGY medium without antibiotics. OD_600_ values were measured every 1 or 2 h.

### Antioxidation Activity Measurements

2′,7′-dichlorofluorescein diacetate (DCFH-DA) was used as a molecular probe hydrolysis to generate DCFH, ROS can oxidize DCFH to generate DCF with fluorescence, which can be measured using a fluorescence spectrometer (SpectraMax M5, United States) with an excitation wavelength of 485 nm and emission wavelength of 525 nm. *D. radiodurans* R1 and the mutant strains were grown to OD_600_ = 1.0 and washed three times with PBS buffer. Pellets were incubated with DCFH-DA at 37°C for 30 min. After incubation, cells were washed three times with PBS buffer and resuspended in 2 mL PBS buffer, and then 1 ml sample was treated with 0, 40, and 80 mM H_2_O_2_ for 30 min, respectively. The accumulation of ROS was measured the manufacturer’s protocol (Beyotime Biotechnology, China).

### Dimethyl Sulfoxide Analyses

DMSO analyses were performed as described previously (Cao et al., 2015). In brief, the strains were grown aerobically to OD_600_ = 1.0. For anaerobic growth, the strains were transferred to TGY medium containing 100 mM DMSO as a terminal electron acceptor at 30°C for 4 days.

### Dimethyl Sulfoxide Reductase Activity Assay

DMSO reductase activity assay was performed as described previously (Miranda et al., 2011). A total of 250 mL of TGY cultures of each strain were grown to OD_600_ = 1.0, harvested by centrifugation, washed in 15 mL buffer A (50 mM Tris-HCl, pH 7.5; 1 mM EDTA, pH 8.0; 2 M NaCl), resuspended in 20 mL buffer A, and lysed by ultrahigh pressure homogenizer (Shanghailitu, China), successively. Cell lysates were clarified by centrifugation (15,000 rpm, 30 min), and protein concentrations were measured using the Bradford assay kit (Beyotime Biotechnology, China). The DMSO reductase activity was monitored at A_600nm_ (15 s intervals for 3.5 min). Assays (4 mL) included cell lysate (1–1.5 mg protein), 0.3 mM methyl viologen in buffer A, and the top filled with nitrogen. The mixture was titrated to 1–1.2 A_600nm_ units with fresh 20 mM odium dithionite (Na_2_S_2_O_4_) in 20 mM sodium bicarbonate (NaHCO_3_) prior to the addition of 10 mM DMSO. One unit (U) of enzyme activity was defined as 1-μm substrate consumed per minute at room temperature, with an extinction coefficient A_600nm_ of 13.6 (mM^–1^⋅cm^–1^) for methyl viologen.

## Data Availability Statement

The original contributions presented in the study are included in the article/Supplementary Material, further inquiries can be directed to the corresponding author/s.

## Author Contributions

YH conceived the project. JC, CP, LW, and YH designed the experiments and drafted the manuscript. JC constructed the vectors and mutants and purified the proteins. CP was responsible for qRT-PCR, enzyme activity, and phenotype analysis. JC, BT, HX, and YZ participated in the data analysis. All authors reviewed the manuscript and approved the version to be published.

## Conflict of Interest

The authors declare that the research was conducted in the absence of any commercial or financial relationships that could be construed as a potential conflict of interest.

## Publisher’s Note

All claims expressed in this article are solely those of the authors and do not necessarily represent those of their affiliated organizations, or those of the publisher, the editors and the reviewers. Any product that may be evaluated in this article, or claim that may be made by its manufacturer, is not guaranteed or endorsed by the publisher.

## References

[B1] Abreu I. A. Hearn A. An H. Nick H. S. Silverman D. N. Cabelli D. E. (2008). The kinetic mechanism of manganese-containing superoxide dismutase from *Deinococcus radiodurans*: a specialized enzyme for the elimination of high superoxide concentrations. *Biochemistry* 47 2350–2356. 10.1021/bi7016206 18247479

[B2] Altmann E. Erbel P. Renatus M. Schaefer M. Schlierf A. Druet A. (2017). Azaindoles as zinc-binding small-molecule inhibitors of the JAMM protease CSN5. *Angew. Chem. Int. Ed. Engl.* 56 1294–1297. 10.1002/anie.201608672 27981705

[B3] Amerik A. Y. Hochstrasser M. (2004). Mechanism and function of deubiquitinating enzymes. *Biochim. Biophys. Acta* 1695 189–207. 10.1016/j.bbamcr.2004.10.003 15571815

[B4] Bischof J. C. He X. M. (2005). Thermal stability of proteins. *Cell Injury Mech. Resp. Rep.* 1066 12–33. 10.1196/annals.1363.003 16533916

[B5] Bueno A. N. Shrestha R. K. Ronau J. A. Babar A. Sheedlo M. J. Fuchs J. E. (2015). Dynamics of an Active-Site Flap contributes to catalysis in a JAMM Family Metallo Deubiquitinase. *Biochemistry* 54 6038–6051. 10.1021/acs.biochem.5b00631 26368668 PMC4962790

[B6] Cao S. Engilberge S. Girard E. Gabel F. Franzetti B. Maupin-Furlow J. A. (2017). Structural insight into Ubiquitin-Like protein recognition and oligomeric states of JAMM/MPN(+) proteases. *Structure* 25 823–833 e826. 10.1016/j.str.2017.04.002 28479062 PMC5831132

[B7] Cao S. Hepowit N. Maupin-Furlow J. A. (2015). Ubiquitin-like protein SAMP1 and JAMM/MPN+ metalloprotease HvJAMM1 constitute a system for reversible regulation of metabolic enzyme activity in Archaea. *PLoS One* 10:e0128399. 10.1371/journal.pone.0128399 26010867 PMC4443979

[B8] Cope G. A. Suh G. S. Aravind L. Schwarz S. E. Zipursky S. L. Koonin E. V. (2002). Role of predicted metalloprotease motif of Jab1/Csn5 in cleavage of Nedd8 from Cul1. *Science* 298 608–611. 10.1126/science.1075901 12183637

[B9] Dahl J. U. Radon C. Buhning M. Nimtz M. Leichert L. I. Denis Y. (2013). The sulfur carrier protein TusA has a pleiotropic role in *Escherichia coli* that also affects molybdenum cofactor biosynthesis. *J. Biol. Chem.* 288 5426–5442. 10.1074/jbc.M112.431569 23281480 PMC3581435

[B10] Dai J. Gao K. Yao T. Lu H. Zhou C. Guo M. (2020). Late embryogenesis abundant group3 protein (DrLEA3) is involved in antioxidation in the extremophilic bacterium *Deinococcus radiodurans*. *Microbiol. Res.* 240:126559. 10.1016/j.micres.2020.126559 32721821

[B11] Dai S. Jin Y. Li T. Weng Y. Xu X. Zhang G. (2018). DR1440 is a potential iron efflux protein involved in maintenance of iron homeostasis and resistance of *Deinococcus radiodurans* to oxidative stress. *PLoS One* 13:e0202287. 10.1371/journal.pone.0202287 30106993 PMC6091924

[B12] Daly M. J. (2012). Death by protein damage in irradiated cells. *DNA Repair* 11 12–21. 10.1016/j.dnarep.2011.10.024 22112864

[B13] Daly M. J. Gaidamakova E. K. Matrosova V. Y. Vasilenko A. Zhai M. Venkateswaran A. (2004). Accumulation of Mn(II) in, *Deinococcus radiodurans* facilitates gamma-radiation resistance. *Science* 306 1025–1028. 10.1126/science.1103185 15459345

[B14] Davies C. W. Paul L. N. Kim M. I. Das C. (2011). Structural and thermodynamic comparison of the catalytic domain of AMSH and AMSH-LP: nearly identical fold but different stability. *J. Mol. Biol.* 413 416–429. 10.1016/j.jmb.2011.08.029 21888914 PMC3321355

[B15] Echalier A. Pan Y. B. Birol M. Tavernier N. Pintard L. Hoh F. (2013). Insights into the regulation of the human COP9 signalosome catalytic subunit, CSN5/Jab1. *Proc. Natl. Acad. Sci. U.S.A.* 110 1273–1278. 10.1073/pnas.1209345110 23288897 PMC3557056

[B16] Galej W. P. Nguyen T. H. D. Newman A. J. Nagai K. (2014). Structural studies of the spliceosome: zooming into the heart of the machine. *Curr. Opin. Struct. Biol.* 25 57–66. 10.1016/j.sbi.2013.12.002 24480332 PMC4045393

[B17] Goswami M. Mangoli S. H. Jawali N. (2006). Involvement of reactive oxygen species in the action of ciprofloxacin against *Escherichia coli*. *Antimicrob. Agents Chemother.* 50 949–954. 10.1128/AAC.50.3.949-954.2006 16495256 PMC1426460

[B18] He Y. Wang Y. Qin C. Xu Y. Cheng K. Xu H. (2020). Structural and functional characterization of a unique AP endonuclease from *Deinococcus radiodurans*. *Front. Microbiol.* 11:1178. 10.3389/fmicb.2020.01178 33117296 PMC7548837

[B19] Hepowit N. L. Uthandi S. Miranda H. V. Toniutti M. Prunetti L. Olivarez O. (2012). Archaeal JAB1/MPN/MOV34 metalloenzyme (HvJAMM1) cleaves ubiquitin-like small archaeal modifier proteins (SAMPs) from protein-conjugates. *Mol. Microbiol.* 86 971–987. 10.1111/mmi.12038 22970855 PMC3558616

[B20] Holmgren A. (2000). Antioxidant function of thioredoxin and glutaredoxin systems. *Antioxid. Redox Signal.* 2 811–820. 10.1089/ars.2000.2.4-811 11213485

[B21] Humbard M. A. Miranda H. V. Lim J. M. Krause D. J. Pritz J. R. Zhou G. (2010). Ubiquitin-like small archaeal modifier proteins (SAMPs) in *Haloferax volcanii*. *Nature* 463 54–60. 10.1038/nature08659 20054389 PMC2872088

[B22] Jeong S. W. Jung J. H. Kim M. K. Seo H. S. Lim H. M. Lim S. (2016). The three catalases in *Deinococcus radiodurans*: only two show catalase activity. *Biochem. Biophys. Res. Commun.* 469 443–448. 10.1016/j.bbrc.2015.12.017 26692481

[B23] Kyuuma M. Kikuchi K. Kojima K. Sugawara Y. Sato M. Mano N. (2006). AMSH, an ESCRT-III associated enzyme, deubiquitinates cargo on MVB/late endosomes. *Cell Struct. Funct.* 31 159–172. 10.1247/csf.06023 17159328

[B24] Leimkuhler S. (2020). The biosynthesis of the molybdenum cofactors in *Escherichia coli*. *Environ. Microbiol.* 22 2007–2026. 10.1111/1462-2920.15003 32239579

[B25] Leimkuhler S. Wuebbens M. M. Rajagopalan K. V. (2001). Characterization of *Escherichia coli* MoeB and its involvement in the activation of molybdopterin synthase for the biosynthesis of the molybdenum cofactor. *J. Biol. Chem.* 276 34695–34701. 10.1074/jbc.M102787200 11463785

[B26] Li S. Cai J. Lu H. Mao S. Dai S. Hu J. (2019). N (4)-cytosine DNA methylation is involved in the maintenance of genomic stability in *Deinococcus radiodurans*. *Front. Microbiol.* 10:1905. 10.3389/fmicb.2019.01905 31497001 PMC6712171

[B27] Lipton M. S. Pasa-Tolic L. Anderson G. A. Anderson D. J. Auberry D. L. Battista K. R. (2002). Global analysis of the *Deinococcus radiodurans* proteome by using accurate mass tags. *Proc. Natl. Acad. Sci. U.S.A.* 99 11049–11054. 10.1073/pnas.172170199 12177431 PMC129300

[B28] Love K. R. Catic A. Schlieker C. Ploegh H. L. (2007). Mechanisms, biology and inhibitors of deubiquitinating enzymes. *Nat. Chem. Biol.* 3 697–705. 10.1038/nchembio.2007.43 17948018

[B29] Makarova K. S. Aravind L. Wolf Y. I. Tatusov R. L. Minton K. W. Koonin E. V. (2001). Genome of the extremely radiation-resistant bacterium *Deinococcus radiodurans* viewed from the perspective of comparative genomics. *Microbiol. Mol. Biol. Rev.* 65 44–79. 10.1128/MMBR.65.1.44-79.2001 11238985 PMC99018

[B30] McCullough J. Clague M. J. Urbe S. (2004). AMSH is an endosome-associated ubiquitin isopeptidase. *J. Cell Biol.* 166 487–492. 10.1083/jcb.200401141 15314065 PMC2172215

[B31] McLuskey K. Harrison J. A. Schuttelkopf A. W. Boxer D. H. Hunter W. N. (2003). Insight into the role of *Escherichia coli* MobB in molybdenum cofactor biosynthesis based on the high resolution crystal structure. *J. Biol. Chem.* 278 23706–23713. 10.1074/jbc.M301485200 12682065

[B32] Miralles-Robledillo J. M. Torregrosa-Crespo J. Martinez-Espinosa R. M. Pire C. (2019). DMSO Reductase family: phylogenetics and applications of extremophiles. *Int. J. Mol. Sci.* 20:3349. 10.3390/ijms20133349 31288391 PMC6650914

[B33] Miranda H. V. Nembhard N. Su D. Hepowit N. Krause D. J. Pritz J. R. (2011). E1-and ubiquitin-like proteins provide a direct link between protein conjugation and sulfur transfer in Archaea. *Proc. Natl. Acad. Sci. U.S.A.* 108 4417–4422. 10.1073/pnas.1018151108 21368171 PMC3060232

[B34] Moretti J. Chastagner P. Gastaldello S. Heuss S. F. Dirac A. M. Bernards R. (2010). The translation initiation factor 3f (eIF3f) exhibits a deubiquitinase activity regulating notch activation. *PLoS Biol.* 8:e1000545. 10.1371/journal.pbio.1000545 21124883 PMC2990700

[B35] Narrandes N. C. Machowski E. E. Mizrahi V. Kana B. D. (2015). Cleavage of the moaX-encoded fused molybdopterin synthase from *Mycobacterium tuberculosis* is necessary for activity. *BMC Microbiol.* 15:22. 10.1186/s12866-015-0355-2 25651977 PMC4326299

[B36] Neumann M. Mittelstadt G. Seduk F. Iobbi-Nivol C. Leimkuhler S. (2009). MocA is a specific cytidylyltransferase involved in molybdopterin cytosine dinucleotide biosynthesis in *Escherichia coli*. *J. Biol. Chem.* 284 21891–21898. 10.1074/jbc.M109.008565 19542235 PMC2755913

[B37] Nijman S. M. B. Luna-Vargas M. P. A. Velds A. Brummelkamp T. R. Dirac A. M. G. Sixma T. K. (2005). A genomic and functional inventory of deubiquitinating enzymes. *Cell* 123 773–786. 10.1016/j.cell.2005.11.007 16325574

[B38] Patterson-Fortin J. Shao G. Bretscher H. Messick T. E. Greenberg R. A. (2010). Differential regulation of JAMM domain deubiquitinating enzyme activity within the RAP80 complex. *J. Biol. Chem.* 285 30971–30981. 10.1074/jbc.M110.135319 20656689 PMC2945588

[B39] Sharon M. Mao H. Boeri Erba E. Stephens E. Zheng N. Robinson C. V. (2009). Symmetrical modularity of the COP9 signalosome complex suggests its multifunctionality. *Structure* 17 31–40. 10.1016/j.str.2008.10.012 19141280

[B40] Shigi N. (2012). Posttranslational modification of cellular proteins by a ubiquitin-like protein in bacteria. *J. Biol. Chem.* 287 17568–17577. 10.1074/jbc.M112.359844 22467871 PMC3366818

[B41] Solomon P. S. Shaw A. L. Young M. D. Leimkuhler S. Hanson G. R. Klipp W. (2000). Molybdate-dependent expression of dimethylsulfoxide reductase in *Rhodobacter capsulatus*. *FEMS Microbiol. Lett.* 190 203–208. 10.1111/j.1574-6968.2000.tb09287.x 11034280

[B42] Wilkinson K. D. (1997). Regulation of ubiquitin-dependent processes by deubiquitinating enzymes. *FASEB J.* 11 1245–1256. 9409543 10.1096/fasebj.11.14.9409543

[B43] Wuebbens M. M. Rajagopalan K. V. (1995). Investigation of the early steps of molybdopterin biosynthesis in *Escherichia*-Coli through the use of *in-vivo* labeling studies. *J. Biol. Chem.* 270 1082–1087. 10.1074/jbc.270.3.1082 7836363

[B44] Yang P. Chen Z. Shan Z. Ding X. Liu L. Guo J. (2014). Effects of FMN riboswitch on antioxidant activity in *Deinococcus radiodurans* under H(2)O(2) stress. *Microbiol. Res.* 169 411–416. 10.1016/j.micres.2013.09.005 24103862

[B45] Yang Y. M. Won Y. B. Ji C. J. Kim J. H. Ryu S. H. Ok Y. H. (2018). Cleavage of molybdopterin synthase MoaD-MoaE linear fusion by JAMM/MPN(+) domain containing metalloprotease DR0402 from *Deinococcus radiodurans*. *Biochem. Biophys. Res. Commun.* 502 48–54. 10.1016/j.bbrc.2018.05.117 29777693

[B46] Zeqiraj E. Tian L. Piggott C. A. Pillon M. C. Duffy N. M. Ceccarelli D. F. (2015). Higher-order assembly of BRCC36-KIAA0157 is required for DUB activity and biological function. *Mol. Cell* 59 970–983. 10.1016/j.molcel.2015.07.028 26344097 PMC4579573

[B47] Zhou M. Sandercock A. M. Fraser C. S. Ridlova G. Stephens E. Schenauer M. R. (2008). Mass spectrometry reveals modularity and a complete subunit interaction map of the eukaryotic translation factor eIF3. *Proc. Natl. Acad. Sci. U.S.A.* 105 18139–18144. 10.1073/pnas.0801313105 18599441 PMC2587604

[B48] Zhou W. H. Liu J. W. (2018). Multi-metal-dependent nucleic acid enzymes. *Metallomics* 10 30–48. 10.1039/c7mt00268h 29094140

[B49] Zupok A. Iobbi-Nivol C. Mejean V. Leimkuhler S. (2019). The regulation of Moco biosynthesis and molybdoenzyme gene expression by molybdenum and iron in bacteria. *Metallomics* 11 1602–1624. 10.1039/c9mt00186g.a 31517366

