# Supplementary material for: DRJAMM Is Involved in the Oxidative Resistance in Deinococcus radiodurans
Source: Front Microbiol. 2022 Jan 28;12:756867. doi: 10.3389/fmicb.2021.756867 (PMC8832034; doi:10.3389/fmicb.2021.756867)
Supplement: Supplementary file 1 [file Data_Sheet_1.docx]

Supporting information

# DRJAMM is involved in the oxidative resistance in *Deinococcus radiodurans*

**Supplementary Table 1.** Strains and plasmids used in this experiment

| Strains | Relevant characteristics | Reference or source |
| --- | --- | --- |
| *D. radiodurans* |  |  |
| DraR1 wt | Wild-type strain ATCC13939 | Laboratory stock |
| *Δdr_0402* | R1 but *dr_0402::str* | This study |
| *Δdr_2607* | R1 but *dr_2607::str* | This study |
| *Δdr_0402_Cwt* | *Δdr_0402* but pRADK*::dr_0402* | This study |
| *Escherichia coli* |  |  |
| DH5α | Cloning strain | TransGen |
| BL21 (DE3) | Expression strain | TransGen |
| plasmids |  |  |
| pET28a | T7 promoter, T7 terminator, Kanr, 6×His-tag coding sequence | Novagen |
| PRADK | *E. coli-D. radiodurans* shuttle vector | Laboratory stock |
| pRADK-*dr_0402* | pRADK*::dr_0402* | This study |

**Supplementary Table 2.** Primers used in this experiment

| Primers | Sequence (5′-3′) |
| --- | --- |
| Expression of proteins | |
| *dr_0402*-F | CCGCGCGGCAGCCATATGGTGCTCCTGACCCTGCCTG |
| *dr_0402*-R | GAGCTCGAATTCGGATCCTCAATTGCTCTCATCGGCG |
| *dr_2607*-F | TGCCGCGCGGCAGCCATATGATGCAGGTGCGCGCCGTG |
| *dr_2607*-R | CGGAGCTCGAATTCGGATCCTCACAGCGTGTCGTGGCC |
| Construction and complement of mutant stains | |
| *Δdr_0402-*p1 | GCCGCACCTTCTCATACGGTT |
| *Δdr_0402-*p2 | CGGGATCCCGGCCCGGCATTGTAAGGACGTT |
| *Δdr_0402-*p3 | CCAAGCTTGGTAAAAGAATGGCCTTGCACTGTTTG |
| *Δdr_0402-*p4 | AGCACGCGGTGGACTTCATCAT |
| *Δdr_0402-*p5 | GCAGGTGCAGGCCCACG |
| *Δdr_0402-*p6 | AATCAGGTAGGGCACCGGGTAG |
| *Δdr_0402_Cwt-*F | CCTGCAGGTCGAATCGGATCCGTGCTCCTGACCCTGCCTG |
| *Δdr_0402_Cwt-*R | CTCACAGGAGGACCCCATATGTCAATTGCTCTCATCGGCG |
| Real time PCR primers | |
| *dr_0402*-RT-F | CTCACAGGAGGACCCCATATGTCAA |
| *dr_0402*-RT-R | TCGGCGAGGTATTCGCGCTCC |
| *dr_2607*-RT-F | TGAGGTGGGCCTGGAAGAACT |
| *dr_2607*-RT-R | TCACGCGGCAGTGGGTGT |
| *dr_0397*-RT-F | TTGATTGAGTGGCCCCGAGAT |
| *dr_0397*-RT-R | GTAGTGGACCGTCTTGGCACAG |
| *dr_1343-*RT-F | GAAAGTAGGCATCAACGGCTTT |
| *dr_1343-*RT-R | TCCACGGTGCCGTCAAAG |


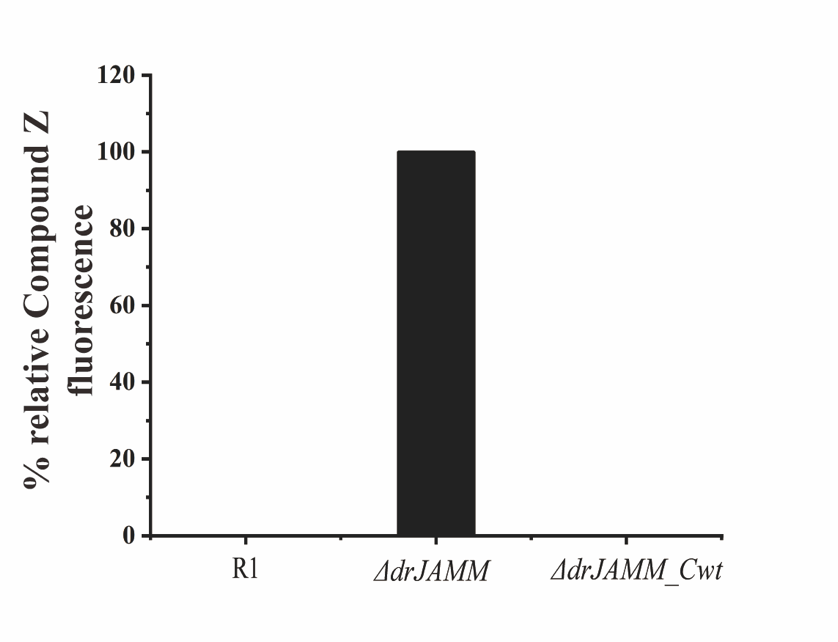
**Supplementary Figure 1.** Analysis of cPMP concentration by determining its stable fluorescent derivative, compound Z, in wild-type strain (R1), the mutant *ΔdrJAMM* (*Δdr_0402*), and *ΔdrJAMM* compensatory stain (*Δdr_0402_Cwt*). The strains were cultured to OD_600_ = 1.0 at 30°C, harvested by centrifugation, and resuspended in ddH_2_O. The lysate was obtained by ultrahigh pressure homogenizer (Shanghailitu, China). cPMP was converted to its fluorescent derivative, compound Z, by adjusting the pH to 2.5 with HCl and incubating with the addition of 200 μl of 1% (w/v) I_2_, 2% (w/v) KI for 14 h. Excess iodine was removed by addition of 110 μl of 1% (w/v) ascorbic acid, and the samples were adjusted to pH 8.3 using 1 M Tris. Compound Z was further purified on Q ion exchange column (5 ml, GE Healthcare Biosciences, USA). After washing with 150 ml of H_2_O and 350 ml of 0.01 N acetic acid, compound Z was eluted from each column with 0.01 N HCl. The fluorescent fractions were freeze-dried, and analyzed by subsequent injection (100 μl) onto a C18 reversed phase HPLC column equilibrated with 10 mM potassium phosphate (pH 3.0) with 1% methanol at an isocratic flow rate of 1 ml/min. The fluorescence was monitored with excitation at 383 nm and emission at 450 nm by liquid chromatograph (Shimadzu, Japan). The amount of compound Z of the *ΔdrJAMM* strain was set to 100%.


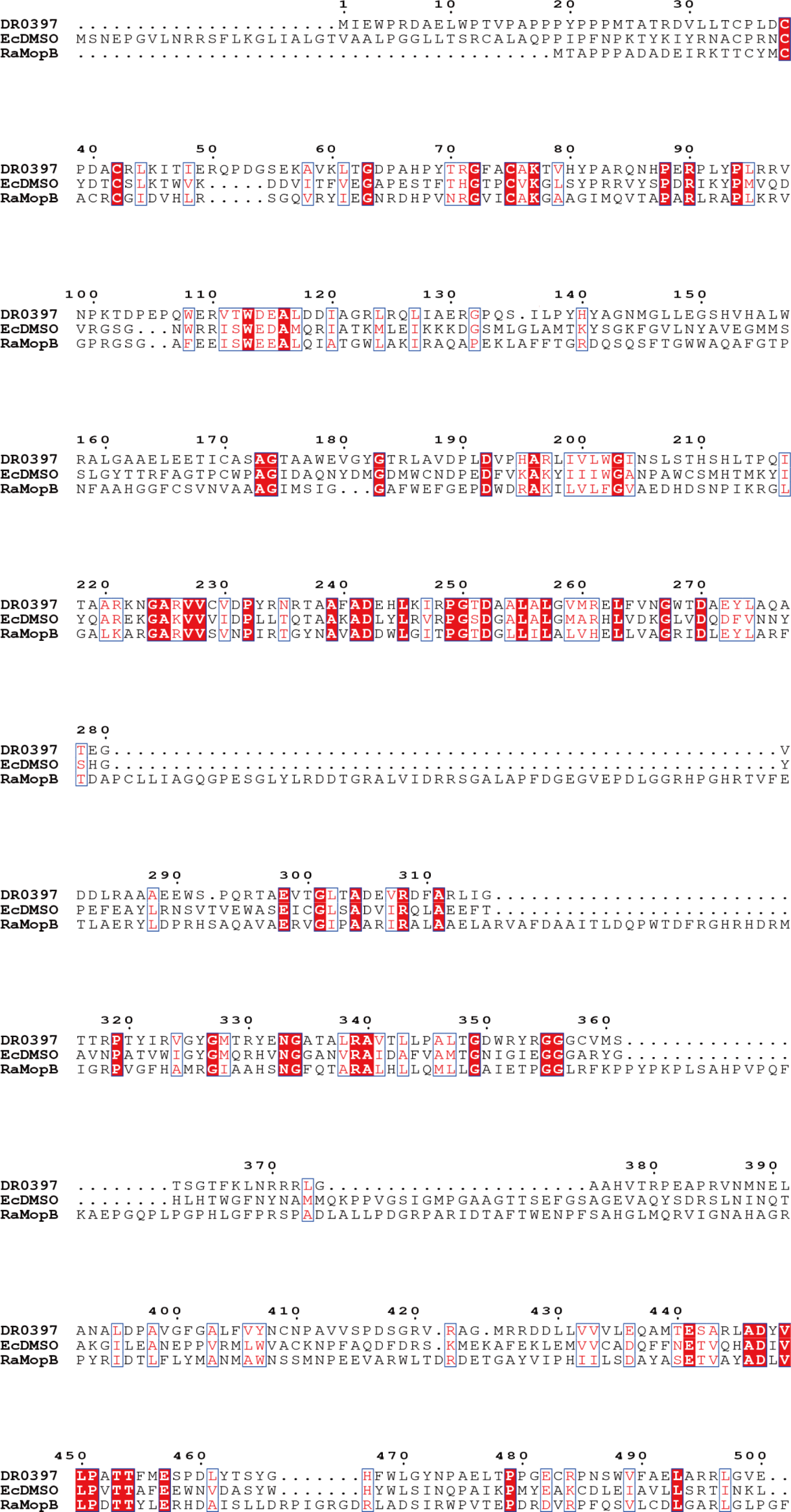


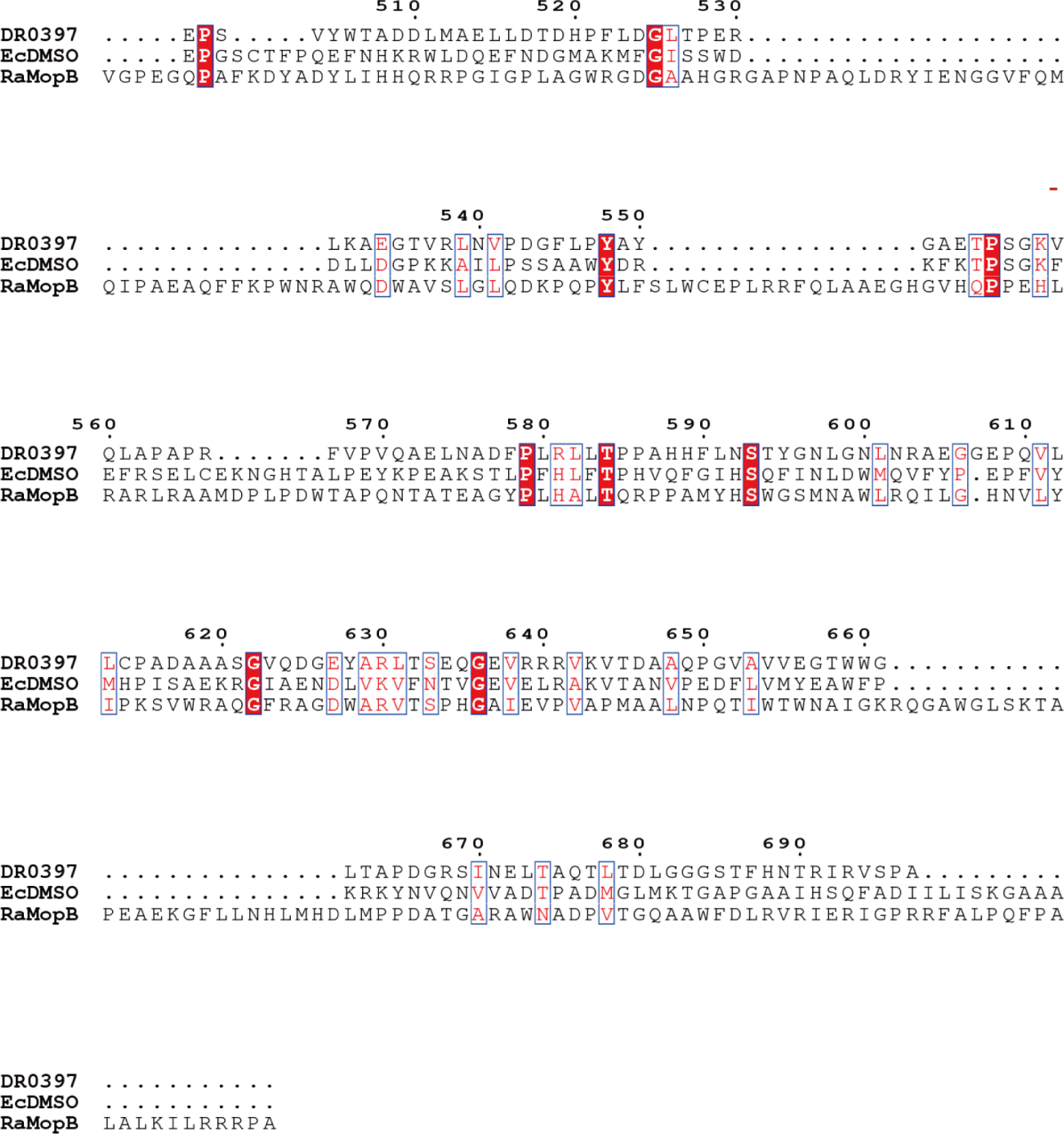


**Supplementary Figure 2.** Sequence alignments among DR0397 and other representative DMSO reductase. DR0397, *Deinococcus radiodurans*; EcDMSO, *Escherichia coli*; RaMopB, *Rhodobacter capsulatu*s.


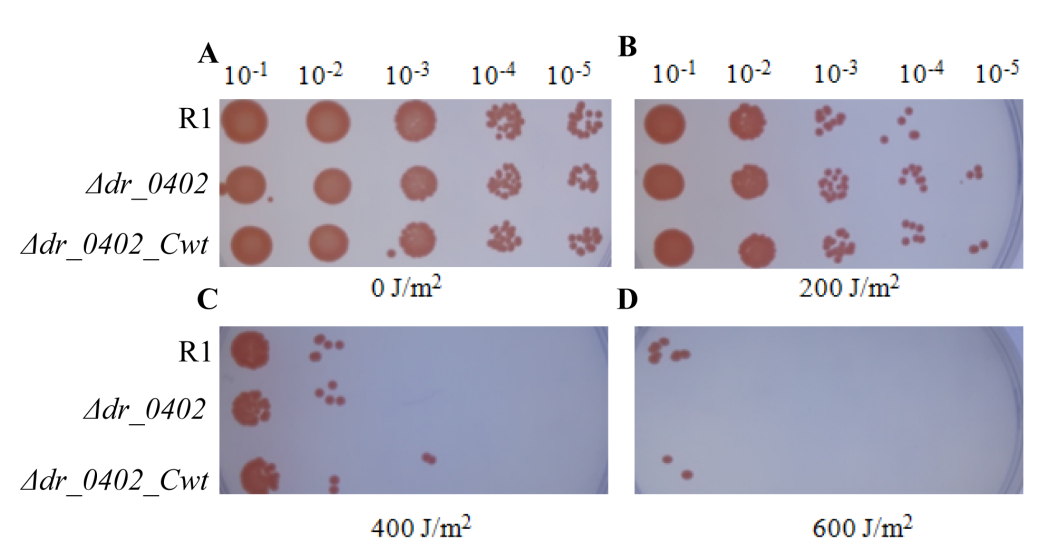


**Supplementary Figure 3.** Phenotypes of the *Δdr_0402* mutant and complementary strains under UV treatment. R1, *Δdr_0402* and *Δdr_0402_Cwt* with OD_600_=0.6 were treated with UV (0-600J/m^2^), respectively, which is eventually spotted onto the TGY plates.
